# Mechanisms of dispersal and colonisation in a wind-borne cereal pest, the haplodiploid wheat curl mite

**DOI:** 10.1038/s41598-021-04525-9

**Published:** 2022-01-11

**Authors:** Alicja Laska, Anna Przychodzka, Ewa Puchalska, Mariusz Lewandowski, Kamila Karpicka-Ignatowska, Anna Skoracka

**Affiliations:** 1 Population Ecology Lab, Institute of Environmental Biology, Faculty of Biology, Adam Mickiewicz University, Uniwersytetu Poznańskiego 6, 61-614 Poznań, Poland; 2 Center for Advanced Technology, Adam Mickiewicz University, Uniwersytetu Poznańskiego 10, 61-614 Poznań, Poland; 3 Section of Applied Entomology, Department of Plant Protection, Institute of Horticultural Sciences, Warsaw University of Life Sciences-SGGW, Nowoursynowska 159, 02-776 Warsaw, Poland

**Keywords:** Ecology, Ecology, Invasive species

## Abstract

Dispersal and colonisation determine the survival and success of organisms, and influence the structure and dynamics of communities and ecosystems in space and time. Both affect the gene flow between populations, ensuring sufficient level of genetic variation and improving adaptation abilities. In haplodiploids, such as *Aceria tosichella* (wheat curl mite, WCM), a population may be founded even by a single unfertilised female, so there is a risk of heterozygosity loss (i.e. founder effect). It may lead to adverse outcomes, such as inbreeding depression. Yet, the strength of the founder effect partly depends on the genetic variation of the parental population. WCM is an economically important pest with a great invasive potential, but its dispersal and colonisation mechanisms were poorly studied before. Therefore, here we assessed WCM dispersal and colonisation potential in relation to the genetic variation of the parental population. We checked whether this potential may be linked to specific pre-dispersal actions (e.g. mating before dispersal and collective behaviour). Our study confirms that dispersal strategies of WCM are not dependent on heterozygosity in the parental population, and the efficient dispersal of this species depends on collective movement of fertilised females.

## Introduction

Successful dispersal allows the colonisation of new habitats and territories, and both processes play an essential role in shaping the demography and genetic diversity of populations. Consequently, these processes contribute to maintenance of population connectivity and persistence in fragmented landscapes and heterogeneous environments^1–3^. They both are important for mitigating extinction risk^2,4,5^ and ensuring a sufficient level of genetic diversity within and between populations, allowing the populations to grow without negative effects of reduced genetic variability^6,7^. Thus, dispersal and colonisation are fundamental biological processes that determine the survival and success of organisms, as well as affect the structure and dynamics of communities and ecosystems in space and time.

To colonise new habitats and territories successfully, organisms need to undertake a proper decision about emigration time, survive during the transfer phase, and settle in an appropriate place, where they must cope with the new environment. Establishing in a place suitable in terms of required abiotic conditions and food resources is not enough to initiate a new population. Organisms also need to deal successfully with competitors, predators, and diseases^8–10^. Altogether, the successful colonisation depends on both the traits and conditions allowing survival during dispersal (including all phases: departure, transfer, and settlement) as well as traits and conditions allowing individuals (dispersers) to survive and reproduce after arrival to a new place^8–11^. Dispersal is particularly risky for passively dispersing organisms, e.g. those carried via wind or water currents, because the transfer and settlement phases of movement are out of their control. Therefore, a question arises about how individuals decrease this uncontrollable way of passive movement and increase the probability of successful colonisation.

The effective establishment of a new population requires a sufficient level of genetic variation of founding individuals^12–17^. In the extreme case of haplodiploid organisms, where even a unfertilized female alone is able to establish the population, the genetic variation of the founding population may depend on the decision of the founder to be fertilised or not. In the latter case, the low number of alleles may lead to adverse effects, such as inbreeding depression^18^. On the other hand, it appears that haplodiploids suffer less from inbreeding depression when compared to diploids, because male haploidy imposes purifying selection on recessive deleterious alleles^19^. Nevertheless, the decrease in overall diversity of alleles (i.e. population gene pool) often happens in haplodiploids and may lead to decreased fitness when the conditions change, as dispersing individuals often experience such situations when they settle in a new area^6,20^. These adverse effects may be, however, reinforced by gene flow, when dispersing individuals land in an area occupied by another subpopulation of conspecifics and reproduce there. However, in the case of unpredictable passive dispersal this strategy is highly unlikely, as dispersers cannot actively choose the place of landing. Therefore, when passively transferred individuals have little opportunity to decide where to land, other mechanisms of inbreeding avoidance are expected. One of the possibilities of maintenance of population heterozygosity would be to increase the variation of immigrants’ gene pool, which allows transferring a sufficiently high proportion of allelic variance of the parental population, enabling the successful establishment of a new population.

The gene pool increase may be ensured by collective dispersal, where a group of individuals is moved together^21,22^. Another possibility is fertilisation before dispersal, as sexual reproduction increases the number of alleles transferred to the new (founding) population and thereby decreases the probability of the reduction of fitness and adaptive potential (which otherwise could lead to population extinction). The effort involved in heterozygosity maintenance (e.g. time and energy spent searching for a partner and mating before dispersal, performing collective behaviour) may differ between populations characterised by various levels of genetic variation. The lower the number of different alleles in a population, the higher the costs associated with finding conspecifics with a different genotype (i.e. kin avoidance). Moreover, inbreeding depression is less harmful for established homozygous populations, which cannot further lose their genetic variation.

In this study we experimentally tested whether collective dispersal and fertilisation before dispersal allow inbreeding avoidance and successful colonisation of passively dispersing, haplodiploid phytophagous mites. Additionally, we checked whether the level of genetic variation of the parental population affects these behavioural and physiological traits. As a study system we chose the wheat curl mite *Aceria tosichella* (Prostigmata: Eriophyidae) MT-1 genotype (known as type 2 in Australia and North America^23,24^; WCM hereafter). WCM is a well-suited model system for studying the mechanisms of dispersal of passively dispersing organisms^25^. It is a tiny mite with a high population growth rate and short developmental time. Its life cycle comprises five stages: egg, larva, quiescent larva, nymph, quiescent nymph, and adult. It is found worldwide and thus easy to collect in the field. Additionally, the mite is easy to rear under laboratory conditions and it quickly reaches high population densities. WCM has a haplodiploid sex-determination system (so-called *arrhenotoky*), where haploid males hatch from unfertilised eggs, whereas diploid females, from fertilised eggs. In this way a single unfertilised female is able to found a new population by producing a haploid son, which after maturing provides sperm, used by females to fertilise eggs and to produce diploid female progeny. The sperm is not transferred with copulation, but instead males produce spermatophores, which are picked-up by females^26^. Because of arrhenotoky, WCM populations are characterised by a female-biased population structure, with females constituting ~ 70% of the total number of individuals^27^. WCM disperses passively with wind currents, and displays some specific behaviours in the presence of wind during the departure stage of dispersal (i.e. chain formation when two or more individuals aggregate by attaching to each other with the use of caudal suckers or legs), suggesting that the collective movement may play an important role in WCM dispersal^28,29^.

To fulfil our aim, we subjected three WCM populations differing in heterozygosity level (low, intermediate, and high; respectively called experimental populations LH, MH, and HH or ‘regimes’ hereafter) to laboratory experiments encompassing passive dispersal via wind currents. Based on assumptions that: (1) collective dispersal increases the chance of transferring different alleles to the founding population and therefore prevents genetic variation loses, and (2) fertilisation before dispersal leads to recombination that increases genetic variation in the founding population, compared to population founded by a virgin female, we expected that pre-dispersal collective behaviour and fertilisation should be a mechanism involved in WCM dispersal and that the studied populations should differ in this regard. Considering an additional assumption that (3) the possibility of inbreeding depression of newly established populations decreases with decreasing source population heterozygosity, as homozygous populations do not suffer any further fitness decrease as a result of heterozygosity loss, we hypothesised that: (a) collective dispersal decreases with the decreasing heterozygosity of parental populations, and (b) the proportion of dispersing fertilised females decreases with the decreasing heterozygosity of parental populations.

## Results

### Genetic differentiation of experimental populations

**Figure 1:**
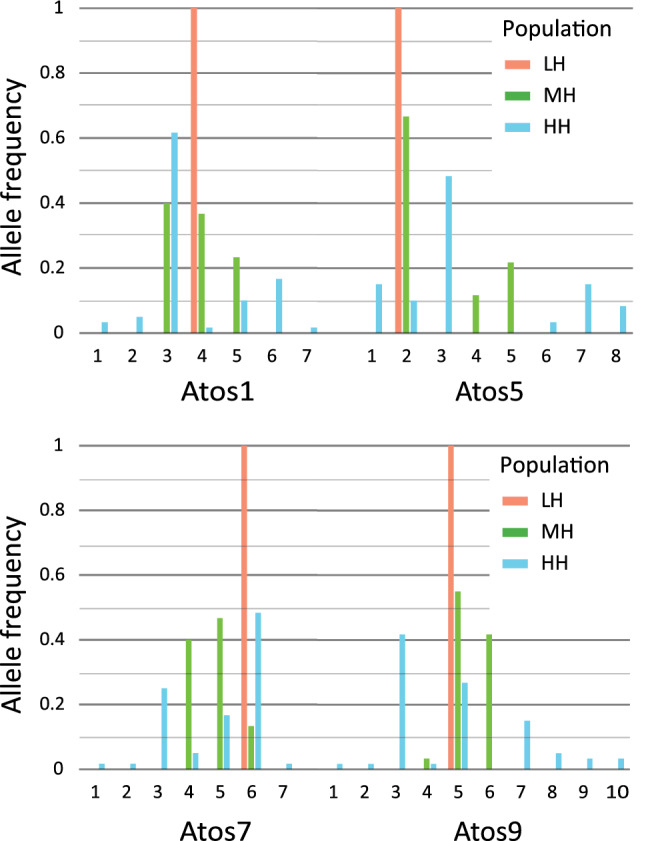
Allele frequency in each tested locus.

Microsatellite loci were genotyped in 90 individuals, 30 from each experimental population. There were significant differences in the number of alleles and in the level of heterozygosity between experimental populations (LH–MH χ^2^ > 94.804, *p* < 0.0001; LH–WH χ^2^ > 104.979; *p* < 0.0001; MH–HH χ^2^ > 102.782, *p* < 0.0001) (Fig. 1). The HH population had a total of 29 alleles, ranging from six to nine per locus, MH had 12 alleles, three variants per locus, and LH had four alleles, with only one allele per locus. Expected heterozygosity (*He*) oscillated between 0.59 and 0.74 for HH, and between 0.66 and 0.50 for MH. Observed heterozygosity (*Ho*) was 0.33–0.57 for HH and 0.03–0.07 for MH (Table 1). Because of the monomorphic allele pattern in the homozygous population LH, both *H*_*E*_ and *H*_*O*_ were 0.

**Table 1:** Characteristics of the four microsatellite loci (Atos1, Atos5, Atos7, Atos9) including the number of alleles (*N*_*A*_), range of allele sizes (range, in bp), expected and observed heterozygosity (*H*_*E*_, *H*_*O*_) and null-allele frequency (NAF) for three experimental populations tested.

	Atos1	Atos5	Atos7	Atos9
**LH population**				
*N*_*A*_	1	1	1	1
Range	164	221	260	255
*H*_*E*_	0.0000	0.0000	0.0000	0.0000
*H*_*O*_	0.0000	0.0000	0.0000	0.0000
NAF	NA	NA	NA	NA
**MH population**				
*N*_*A*_	3	3	3	3
Range	154–166	221–240	248–260	254–257
*He*	0.6621	0.5034	0.6147	0.5316
*Ho*	0.0667	0.0667	0.0667	0.0333
NAF	0.3557	0.3002	0.3386	0.3242
**HH population**				
*N*_*A*_	7	6	7	9
Range	143–187	204–254	231–262	151–286
*He*	0.5876	0.7153	0.6842	0.7395
*Ho*	0.3333	0.5333	0.5667	0.3667
NAF	0.1839	0.2012	0.0933	0.2458

For comparisons between populations, *F*_*ST*_ (genetic variance between populations) was estimated, as it is more intuitive than χ^2^, and can be interpreted as the proportion of the total genetic variance contained in a subpopulation relative to the total genetic variance. Estimates for all loci detected high differentiation between the following populations: 49% (*F*_*ST*_ = 0.4859) between LH vs. MH populations, and 56% (*F*_*ST*_ = 0.5624) between LH and HH populations. The differentiation between MH and HH populations was lower, reaching 24% (*F*_*ST*_ = 0.2398).

As expected, the three experimental populations differed in inbreeding coefficient *F*_*IS*_. In the MH population it was higher than in HH (0.9006 and 0.3437, respectively), whereas all loci in the LH population were homozygous.

### Dispersal

**Figure 2:**
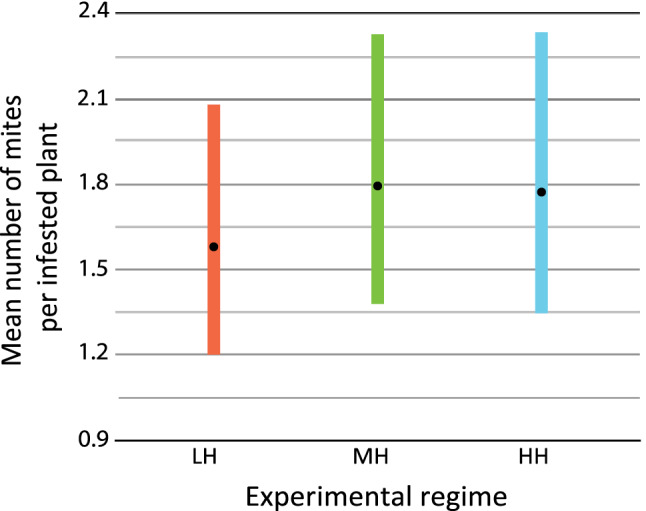
Mean number of mites found on infested plants in each experimental regime. Dots represent means and bars denote 95% CI.

Dispersing individuals settled on only a few of the 20 target wheat shoots per pot in each experimental regime: mean number of inhabited plants with 95% CI for LH: 3.3 (1.94–4.06); for MH: 3.4 (2.53–4.27); for HH: 3.0 (2.25–3.75). The plants occupied by mites were infested on average by more than one individual (χ^2^ = 45.182, *p* < 0.0001); mean number of individuals per inhabited plant with 95% CI for LH: 1.58 (1.20–2.08); for MH: 1.80 (1.38–2.33); for HH: 1.77 (1.35–2.34) and the pattern of settlement did not differ between the tested regimes (χ^2^ = 0.538, *p* = 0.7640) (Fig. 2).

**Figure 3:**
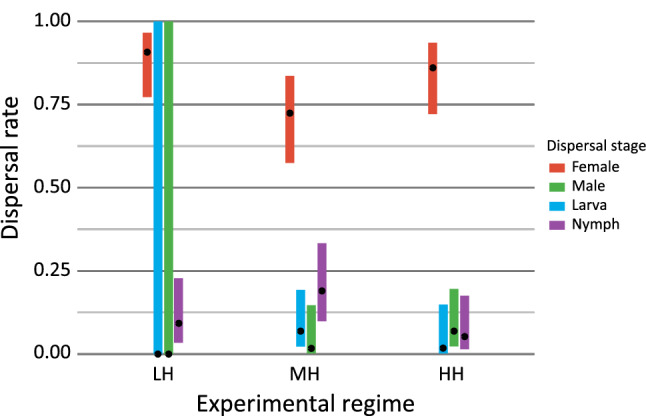
Dispersal rate of different developmental stages in each experimental regime. There were no males and larvae dispersing in the LH colony, so the dispersal rate is predicted between 0 and 1. Dots represent means and bars denote 95% CI.

Adult females dispersed significantly more often than the other developmental stages (Tukey post-hoc comparisons of female dispersal rates vs. all other developmental stages in all experimental populations—all *p* < 0.0001; Fig. 3). Specifically, among all individuals, 49 females dispersed in LH, 42 in MH, and 49 in HH, whereas 5, 16, and 8 individuals of the other stages dispersed in LH, MH, and HH, respectively (Fig. 3). The probability of female dispersal did not differ between regimes (LH–WH: *t* = 0.788, *p* = 0.7192; LH–MH: *t* = 2.592, *p* = 0.0683; MH–WH: *t* = − 1.817, *p* = 0.2185). The proportion of females to total adults among dispersing individuals in each regime was significantly more female-biased than in the respective parental population (χ^2^ = 11.513; *p* = 0.0007) (for details, see “Supplementary Information S1”).

### Colonisation

**Figure 4:**
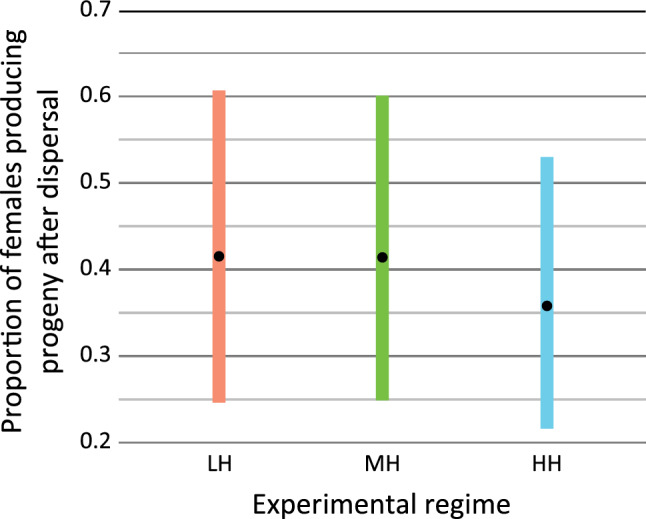
Proportion of females reproducing after dispersal to all dispersing females. Dots represent means and bars denote 95% CI.

Colonisation was studied as a process by which a dispersed WCM female introduces its first generation offspring into a new environment. In total, we found 140 females on target plants, from which 114 were taken for further analysis (26 females did not survive the transfer from the target plants to the experimental area). Out of these 114 females tested, 61 laid eggs. All these females produced at least one female egg (F1), which indicates that they all were inseminated. The proportion of females that laid eggs to all females that dispersed did not differ between experimental regimes (χ^2^ = 0.291, *p* > 0.1; Fig. 4).

**Figure 5:**
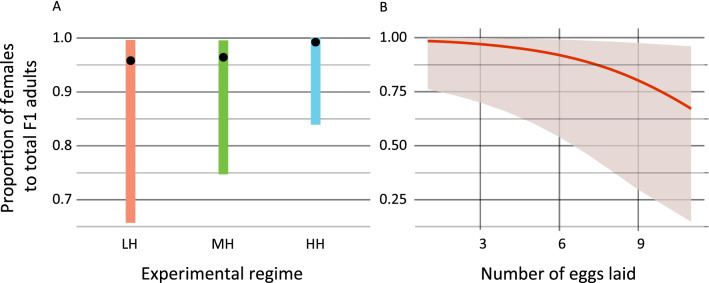
Proportion of females to total adults in the first generation (F1) after dispersal, in relation to (**A**) the level of heterozygosity in the parental population; (**B**) the total number of eggs laid till 4th day after dispersal. Dots (**A**) and line (**B**) represent means, bars (**A**) and shaded area (**B**) denote 95% CI.

The population structure of the F1 generation was female-biased independently of the level of heterozygosity (χ^2^ = 1.269; *p* = 0.530) (Fig. 5A). The overall number of laid eggs significantly influenced the proportion of females to total F1 adults (χ^2^ = 4.861; *p* = 0. 0275). The more eggs a female laid, the more males were in the F1 generation (Fig. 5B).

## Discussion

Colonisation success of a founding population depends on many external factors as well as on biological properties of dispersing individuals, among which the sufficient genetic variation of immigrants is extremely important. Too low genetic variation may result in a fitness decrease caused by the low adaptive abilities of colonisers or by a greater inbreeding depression^18^. A decrease in genetic variation of the founding population may occur when a small group of migrants does not represent genetically the initial population from which it originates^30^. This may result in increased sensitivity to genetic drift, an increase in inbreeding, and therefore lead to relatively low genetic variation of the newly established population^31–33^. The strength of the founder effect and the associated loss of genetic variation may depend on the genetic variation within the parental population from which immigrants derive, as the more homozygous the parental population is, the lower is the chance of transferring many different alleles to the new population.

In this study, we subjected three parental populations of haplodiploid phytophagous wheat curl mite (WCM), differing significantly in their genetic variation (low, intermediate, and high), to passive dispersal with wind. We tested whether parental populations of high or intermediate heterozygosity trigger mechanisms such as collective behaviour and fertilisation before dispersal, to prevent a decrease in genetic variation in the founding population; and whether the low heterozygosity of the parental population results in a decrease in this phenomenon. Contrary to our expectations, we showed that the level of heterozygosity of the parental population does not influence the intensity of pre-dispersal collective behaviour and the proportion of dispersing fertilised females. This suggests that WCM has no costs due to decreasing the genetic variation in the parental population. The phenomenon of similar dispersal success despite the different parental heterozygosity implicates the increased WCM ability to colonise new territories and broadening its distribution ranges. This feature may be one of the major factors influencing the invasive potential of WCM. Our results also show that passive dispersal of WCM is not a stochastic process and adult females are the main dispersal stage and undertake dispersal after specific actions, such as pre-dispersal mating and collective behaviour, regardless of genetic variation of the parental population.

Dispersal is one of the mechanisms avoiding the costs of inbreeding depression due to the chance of joining another subpopulation of conspecifics and reproduce there^34,35^. This case is well known in haplodiploid eusocial Hymenoptera (ants, some bees, and wasps)^36^. In nature, WCMs disperse passively with wind, and thus the place where they land is outside their control. Therefore, the chance of WCM dispersers to join another population after being blown by wind is low. In consequence, other mechanisms of increasing the heterozygosity of populations established after dispersal may be expected. In our experiment, dispersed WCM individuals on newly inhabited plants were found in small groups of joined individuals, regardless of the genetic variation of the parental population (Fig. 2). This confirmed that collective dispersal (forming so-called chains) is a general adaptation to aerial dispersal of WCM. It was previously suggested that dispersal of multiple individuals together increases the level of heterozygosity in founder populations of eriophyoid mites and reduces potential genetic costs of single individual dispersal^36^. The colonisers establishing a new population collectively provide more alleles than a female travelling alone and diminish the adverse founder effect. The evolution of such a mechanism is particularly important in the case of unpredictable and uncertain dispersal, such as transfer with wind. Although this is the most effective way of spreading for many small invertebrates, it is also very risky because the dispersers cannot control the direction of movement and the place of landing as well as they cannot stop the transfer when weather conditions suddenly change^8^. This high level of unpredictability is especially disadvantageous for highly host-specific obligatory phytophagous animals, such as some WCM biotypes. Aerially blown individuals can be transferred to non-host species. Even if they successfully settle on a proper host, it may be uninhabited by conspecifics. Hence the collective dispersal behaviour can reduce the costs of risky aerial dispersal, and such a phenomenon has been previously documented in e.g. haplodiploid phytophagous spider mites^21,22^. However, Yearsley et al.^37^ concluded that collective dispersal may have knock-on consequences for the population genetic structure, because it can reduce the mixing effect of dispersal (even in systems with high rates of migration), which slows down the rate at which related lineages (e.g. siblings) move away from one another. Yet, it is known that in haplodiploid organisms dispersal may be the key mechanism allowing to avoid inbreeding and its consequences^38,39^. The main role of females in WCM dispersal has been suggested already 50 years ago on the basis of dispersing individuals caught in sticky traps in the field^40^. However, that prediction has not been rigorously experimentally confirmed so far, whereas the question of whether fertilised or virgin females disperse has never been examined. Here, we experimentally proved that females disperse significantly more often than other stages (Fig. 3). Moreover, the differences in proportion of females to total adults between parental and disperser populations confirmed that WCM female is, indeed, the main dispersal stage in WCM and that their insemination takes place before departure. This increases the possibility of transferring different alleles to the founding population and prevents heterozygosity losses. Additionally, fertilisation before dispersal allows for producing female eggs just after the settlement and therefore allows faster population multiplication than in a situation when a virgin female first needs to produce male eggs and wait several days for the possibility to be fertilised. Also, fertilisation increases the genetic variation of offspring due to meiotic chromosome recombination. Moreover, female eggs are not exposed to the purging effect, which decreases males’ survival rate and decreases the frequency of recessive alleles in the population. It is noteworthy that when experimental females produced only one egg, the egg was always fertilised, and the F1 offspring was female-biased (Fig. 5A,B). Production of female-biased offspring constituting the first generation in a new environment may have enormous consequences for population persistence. It allows a rapid population growth, which may boost colonisation and increase the invasive potential^41^.

In our study we showed that only half of dispersing females were able to survive and reproduce in a new environment (Fig. 4). Such a decrease in colonisation success may be a result of injuries that some individuals could suffer during dispersal, e.g. dying out due to unfavourable humidity conditions, strong turbulent wind, starvation, high light intensity or environmental pollution. Aerial movement in nature is associated with high energetic and physiological costs resulting from the stressful abiotic factors or restricted access to food resources when dispersal is not successful on the first attempt, and more dispersal efforts are needed to colonise a new habitat successfully. Another possible cause of low colonisation capabilities of some females is that only a fraction of females found on target experimental plants could be true dispersers, well adapted to aerial movement. Such true dispersers in WCM may, for example, have specific physiological or morphological traits protecting them against drying out or allowing more effective dispersal. Morphologically and physiologically different dispersers and residents were distinguished in many species (reviewed in^9^). An example is *Abacarus hystrix*^42^, an eriophyoid mite species associated with grasslands. Individuals of *A. hystrix* enlarge the production of lateral and dorsal bands of wax filaments in summer, thus increasing the total body surface and reducing water loss. Such adaptations play an important role in successful dispersal by increasing mite survival during migration. In our experiment, individuals that were not successful colonisers might have been accidentally blown by wind. This scenario assumes the existence of dispersal syndromes in WCM, which has been already postulated before^29^. A previous study examining the first stage of dispersal, i.e. departure events, showed that individuals being blown by wind differed significantly in their morphology and behaviour from those that did not leave the source patch. Dispersers were more elongated and formed chains, in which they dispersed in specific wind conditions more often than the rest of the population^29^. Distinguishing between these two dispersal phenotypes in WCM could help to understand the covariation of morphological, behavioural, or life-history traits associated with the movement of this mite. The above mentioned and our findings may have an important impact on predicting the intensity, nature, and modalities of WCM spread in nature. It may also help us understand the mechanistic determinants and constraints associated with WCM dispersal. Finally, studying dispersal syndromes may provide information about the proximate motivations and ultimate causes of WCM dispersal^43^.

Altogether, our findings experimentally proved that in WCM: (1) females are the most effective dispersers, colonisers, and population founders; (2) females are adapted to colonisation behaviourally by collective movement and physiologically by being fertilised before departure; (3) dispersal strategies are not dependent on the level of heterozygosity of the parental population, suggesting that WCM has no costs due to inbreeding.

## Material and methods

### Experimental mite populations

Three WCM populations differing in the level of homozygosity were used in the experiment. The first population, characterised by a high level of heterozygosity (HH), was established from WCM individuals collected in July 2017 from nine geographically distant cereal fields in Poland (details in^44^). At each location, ten spikes of common wheat (*Triticum aestivum*) were collected in the field, transported in cold and shaded conditions, and subsequently investigated under a stereomicroscope in the Population Ecology Lab of Adam Mickiewicz University, Poznań, Poland. Several mite specimens from each spike were then barcoded, using a fragment of mitochondrial DNA encoding cytochrome *c* oxidase I (COI), to identify the WCM genotypes. About 1000 mite individuals from 26 populations for which the presence of WCM MT-1 genetic lineage was confirmed were transferred to wheat plants cultivated in one pot to establish a new population and kept together for 3 years before the experiment. The second population, with a moderate level of heterozygosity (MH), was obtained from several dozen mite individuals collected from spikes sampled in one cereal field (Choryń, Poland, GPS: 52.0433, 16.7672; GenBank Acc. No: JF920077) in 2012 and reared without the influx of individuals from other populations until the experiment was conducted. The barcoding procedure identifying the MT-1 lineage was the same as above (details in^45^). The third population, with a very low level of heterozygosity (LH), was established in 2016 from a single female nymph transferred from the MH population to a clean wheat plant. An unfertilised female hatched from the nymph, thus it was able to lay only haploid (i.e. male) eggs, from which males hatched. The unfertilised female picked up a spermatophore laid by one of the males, and subsequently laid diploid female eggs. When the daughter female nymph developed, it was transferred to another clean wheat plant, and this procedure was repeated ten times. In this way we obtained a highly inbred population.

Each experimental population was separately reared on wheat plants under constant laboratory conditions (22–24 °C, 12:12 D/N; 45% RH) in isolators (cages consisting of metal frames wrapped in fine nylon mesh).

### ***Summary of the experimental approach (Fig. ******6******)***

**Figure 6:**
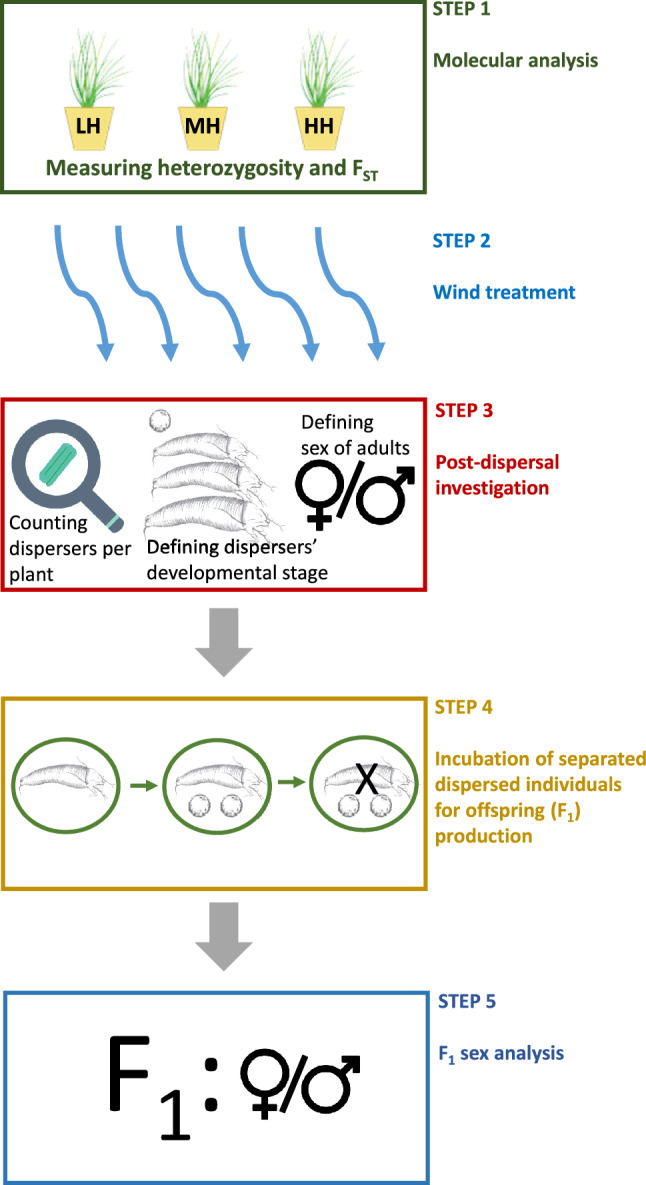
Scheme of the experimental approach.

Step 1: Molecular analysis of the experimental populations—assessing differences in the level of heterozygosity between populations by using microsatellite markers.

Step 2: Experimental dispersal with wind.

Step 3: Post-dispersal investigation.

- Counting the number of dispersers per plant
- Identifying dispersers’ developmental stage
- Identifying the sex of adults

Step 4: Incubation of single dispersed individuals for offspring (F1) production.

Step 5: F1 sex identification.

### Microsatellite loci analysis

To assess differences in the level of homozygosity between experimental populations, molecular analysis using nine microsatellite markers was conducted, following the protocol established by Miller et al*.*^46^, who developed microsatellite markers for Australian WCM populations. For this purpose, 30 mite specimens from each experimental population (HH, MH, LH) were collected and their DNA was extracted. Specimens were transfixed with a dissection needle to prick the cuticle, and placed each in a separate microcentrifuge tube with 20 µl of Mili-Q water. The tubes were centrifuged at 14,100 rcf for 30 s and then 5 µl of Proteinase K (~ 20 mg/ml; Thermo Scientific) and 80 µl of 9% Chelex (Sigma-Aldrich) solution were added. The samples were incubated at 56 °C for 60 min and then at 100 °C for 8 min. After the incubation, the tubes were centrifuged at 9600 rcf for 2 min and 80 µl of the supernatant with mite DNA were transferred to a single new tube. Microsatellite markers were amplified in polymerase chain reaction (PCR) with HotStartTaq Master Mix (Qiagen), a sequence-specific forward primer, with the M13 tail at its 5′end, a sequence-specific reverse primer and the universal fluorescent-labelled M13 primers (Table 2). The 10 µl of PCR mixture included 5 µl of Master Mix, 0.06–0.20 µl of each 10 µM primer, 0.16–0.24 µl of M13 primers, and 3 µl of DNA template. PCR cycling conditions comprised initial denaturation at 95 °C for 5 min; 36 cycles of 95 °C for 30 s, 55 °C for 90 s, and 72 °C for 30 s; and final extension at 65 °C for 30 min. The samples were then diluted with 10 µl of Mili-Q water and genotyped using 3130xl capillary analyser (Genetic Analyzer). Product lengths were determined relative to a GS 600 LIZ size standard. Genotypes were scored using the GeneMapper version 5.0 software (Applied Biosystems) and analysed using GenAlEx 6.5 to assess allele frequency.

**Table 2:** Microsatellite loci and specific primer sequences chosen from Miller et al.^46^ (for this study, fluorescent M13 tails were added to original F-primers).

Locus	Primer sequence 5′–3′
Atos 1	F-ACGAGACGCCTCTAACGTGT R-CCGGAATCTAATTCCCATCC
Atos 5	F-TTCGCCGGAATACACTTGAC R-TGCAGTCAGCACTAGCGTTC
Atos 7	F-ATCAGGCATGCAACAGGTAG R-GTCGCGATCTGTGGTTTCTT
Atos 9	F-GGAGCGAACTTTGTGTCCTT R-GCCCCTATCGACTTTTGTGA

Only four out of nine markers fit the genomes of mites from our experimental populations, probably due to the great geographical distance between Australia and Poland.

### Experimental set-up

To test whether the level of heterozygosity influences WCM dispersal and colonisation ability, we performed the experiment of wind-mediated dispersal in ten replications for each experimental mite population. It was conducted in custom-designed dispersal wind tunnels according to Kuczyński et al.^25^. Each tunnel was composed of: (1) a wind generator producing wind at the speed of 2.5 m/s (sufficient wind speed for WCM MT-1 dispersal^29^); (2) a source plant, i.e. a single wheat plant infested by mites that was exposed to wind to trigger mite dispersal; and (3) target plants, i.e. an area composed of 20 wheat plants to which mites could disperse. Mites were exposed to a fluctuating wind regime to mimic natural conditions in which the wind blows intermittently and allows mites to receive cues (kairomones) from the plant located downwind. The upwind source plants were randomly chosen from each experimental population and transplanted to small pots. All experimental populations were comparable in size. The exact number of mites infesting source plants was estimated carefully under a stereomicroscope to avoid plant damage. From LH, MH, and HH experimental populations, we selected plants infested by a comparable number of WCM individuals. The plants from LH, MH, and HH experimental populations were infested by the following numbers of WCMs: on average by 720 (95% CI 379.76–1060.24), 1220 (95% CI 515.21–1924.79), and 1060 (95% CI 588.85–1531.15) individuals, respectively. Each blowing session lasted 24 h. After exposure to wind currents, the target plants were incubated for 24 h at room temperature to allow mites to hide in leaf sheaths and let those individuals that landed on soil to climb the plants. This step was essential, as it limited the risk of overlooking or losing individuals, what could have happened when doing plant examination immediately after wind treatment. After the incubation, we cut each plant, carefully examined it under the stereomicroscope Olympus SZX10, and counted the number of dispersers. Subsequently, we identified the developmental stage of each individual by its morphology, and transferred it to the fragment of wheat leaf placed in 12-well plates filled with modified in vitro MS medium (according to Karpicka-Ignatowska et al.^47^). Individuals were then incubated at 27 °C, 60% RH, which are optimal conditions for rapid WCM development in laboratory conditions^48^. Females were kept on the leaf fragment for 4 days and were allowed to lay eggs (F1). Other developmental stages were incubated until they developed into adults. Then, adult individuals were incubated for subsequent 4 days and females allowed to lay eggs (F1). Individuals that did not lay eggs were mounted on the Berlese medium in permanent microscopic slides (according to the approach proposed by Amrine and Manson^49^ as well as de Lillo et al.^50^, and their sex was identified under the phase-contrast microscope Olympus BX41.

Leaf fragments with F1 eggs, laid by females that dispersed successfully (as adults or immature stages) were monitored daily, and individuals that reached maturity were mounted on microscopic slides to identify their sex (as above). Therefore, we were able to check whether the female that dispersed was inseminated before it undertook dispersal or it was a virgin. In the former case, the offspring contained females, while in the latter case the offspring was represented solely by males. This experimental procedure allowed us also to assess the proportion of females to total F1 adults (colonisers hereafter).

### Statistical analyses

All statistical analyses were performed in R version 4.0^51^.

#### Genetic differentiation of experimental populations

The expected and observed heterozygosity (*H*_*E*_ and *H*_*O*_), allele frequencies, null allele frequency (NAF), fixation index (*F*_*ST*_), inbreeding index (*F*_*IS*_), and differentiation between the populations were tested using the GenePop R package^52^ based on data collected from four microsatellite loci that were genotyped in 90 individuals, 30 from each experimental population.

#### Dispersal

First, we tested whether mites settled collectively after dispersal using a generalised linear mixed model with the overall number of dispersers as a response, while regime, number of infested plants as well as number of dispersers per plant as predictors. The negative binomial distribution with logit-link function was used. We also used population ID and plant ID as random variables to account for using multiple repetitions from the same population and multiple observations from the same plant. The type II Wald χ^2^ test^53^ was performed to assess the significance of the fixed effects. The model was fitted using the glmmTMB R package^54^.

Second, we tested whether dispersal rate (the proportion of dispersing individuals of the given developmental stage to all dispersers) at each developmental stage depended on the experimental population, by using multinomial log-linear model with the matrix including counts of dispersers per developmental stage as a response and experimental population as a predictor. Next, we performed the Tukey post-hoc analysis to compare how the dispersal pattern of each stage depended on experimental population. The model was fitted using the mgcv R package^55^, while the post-hoc analysis was conducted using the emmeans R package^53,56^.

#### Colonisation

First, we tested whether the proportion of females able to produce progeny, relative to all females that dispersed, depended on the experimental population heterozygosity level using generalised linear mixed model with the females’ ability to produce offspring (coded “0” or “1”) as a response and regime as a predictor. The binomial distribution with logit-link function was used. We used random intercepts defined as mite ID nested in repetition ID to account for using multiple females originating from the same repetitions, and multiple repetitions from the same population. The type II Wald χ^2^ test^53^ was performed to assess the significance of predictors. The model was fitted using the glmmTMB R package^54^.

Second, we tested whether the level of source population heterozygosity influences the proportion of females to total adults among colonisers, using a generalised linear mixed model with the proportion of females as a response, and egg number as well as the experimental population as predictors. The binomial distribution with logit-link function was applied. We used repetition ID nested in mite ID as a random variable to account for using multiple females originating from the same repetitions, and multiple repetitions from the same population. The type II Wald χ^2^ test^53^ was performed to assess the significance of fixed effects. The model was fitted using the glmmTMB R package^54^.

### Ethics declarations

No ethical approval or specific permit was needed for rearing and experimental use of *A. tosichella*, which is neither protected nor endangered. The study adheres to the ASAB/ABS Guidelines for the Use of Animals in Research, the legal requirements of Poland and complied with existing laws regulating the treatment of invertebrates in the EU.

## Supplementary Information

Supplementary Information.

## Data Availability

Data available from Zenodo repository under: https://doi.org/10.5281/zenodo.5537170.
