# Supplementary material for: Mechanisms of dispersal and colonisation in a wind-borne cereal pest, the haplodiploid wheat curl mite
Source: Sci Rep. 2022 Jan 11;12:551. doi: 10.1038/s41598-021-04525-9 (PMC8752673; doi:10.1038/s41598-021-04525-9)
Supplement: Supplementary file 1 — Supplementary Information. [file 41598_2021_4525_MOESM1_ESM.pdf]

Mechanisms of dispersal and colonisation in a wind-borne cereal pest, the haplodiploid wheat curl mite

Alicja Laska[1,2,*], Anna Przychodzka[1], Ewa Puchalska[3], Mariusz Lewandowski[3], Kamila Karpicka-Ignatowska[1], Anna Skoracka[1]

[1]Population Ecology Lab, Institute of Environmental Biology, Faculty of Biology, Adam Mickiewicz University, Uniwersytetu Poznańskiego 6, 61-614 Poznań, Poland

[2]Center for Advanced Technology, Adam Mickiewicz University, Uniwersytetu Poznańskiego 10, 61-614 Poznań, Poland

[3]Section of Applied Entomology, Department of Plant Protection, Institute of Horticultural Sciences, Warsaw University of Life Sciences – SGGW, Nowoursynowska 159, 02-776 Warsaw, Poland

*corresponding author: Alicja Laska, e-mail: alicja.laska@amu.edu.pl

Appendix S1: Proportion of females to total adults in experimental populations

We checked the proportion of females in experimental populations (LH, MH, HH) before and after dispersal. In the first case, we randomly chose 10 infested leaf fragments (2 cm long), then collected all adult mite individuals and mounted them on Berlese medium in permanent slides, subsequently identifying their sex under a phase-contrast microscope (Olympus BX41). We counted the total number of males and females from 10 repetitions per experimental population.

To determine the proportion of females in populations 24 h after dispersal, the developmental stages of dispersers were identified directly by their morphology before transferring them to further incubation (see "Experimental setup", step 3 in the main document for details).

The proportion of females before and after dispersal differed significantly χ^2 =11.5132; p = 0.0007), as the populations after dispersal were more female-biased. This pattern was significant in all experimental regimes (χ^2 = 6.5006, p = 0.0388).

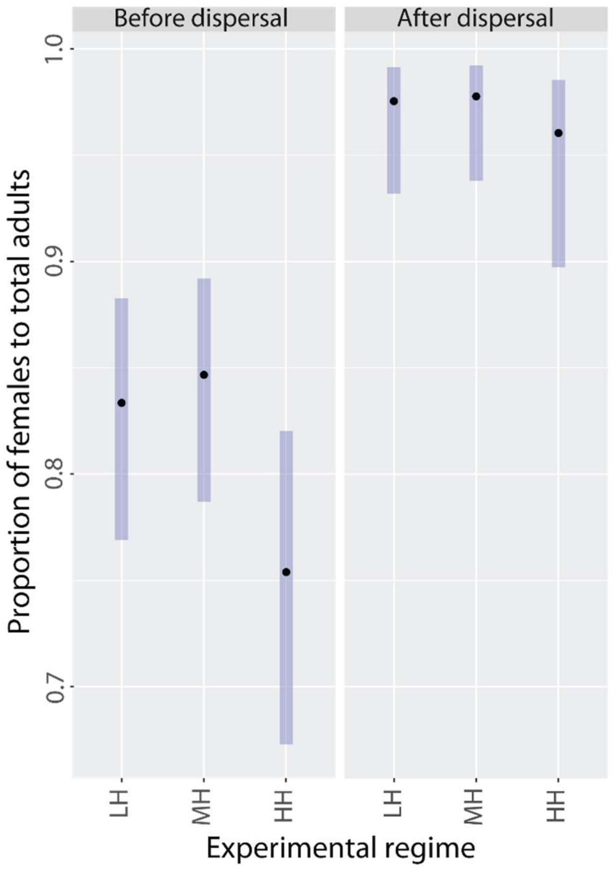

Supplementary Fig. S1 online Proportion of females to total adults in LH, MH and HH populations before and 24 h after dispersal. Dots represent means, while bars denote 95% CI.
